# MED40/472: A WWW Multimedia Textbook of Internal Propedeutics

**DOI:** 10.2196/jmir.1.suppl1.e75

**Published:** 1999-09-19

**Authors:** J Zelenková, J Vejvalka, D Holá, J Segethová

**Affiliations:** ^1^2nd Medical FacultyCharles UniversityPrahaCzech Republic

**Keywords:** Medical Education, Distance Education, Internet, Multimedia, Internal Medicine, Physical Examination

## Abstract

**Introduction:**

Traditional ways of teaching techniques of physical examinations in the first clinical courses are rather demanding in terms of teacher involvement and a pool of patients suitable for demonstrations. For a long time, various audio-visual tools have been used to save teachers' and students' time and patients' patience. The modern technology of WWW publishing of multimedia allows good access to such teaching materials - and there already exist several collections of heart sounds, breath sounds etc. The aim of our project is to design and set up a comprehensive multimedia textbook of internal propedeutics that would present various physiological and pathological findings (auscultation, inspection, basic imaging) in the context of diagnostic patient investigation - the status praesens - as it is taught in the first clinical courses.

**Methods:**

Unlike classical textbooks, hypertext presentation allows to ogranize the material into several structures - reflecting various approaches: systemic (digestive, cardiovascular etc.) approach, nosological, differential diagnoses, etc. To identify and implement the various useful approaches is the most difficult part of the task. The accompanying illustrative material is being prepared with the use of modern technologies - digital camera, scanner, video-camera and digitizer, digital audio recording, etc.

**Results:**

In the first year of the project, the skeleton of the multimedia presentation is being constructed - corresponding to the various approaches to the subject. Concurrently, suitable illustrative material is being gathered from cases of the Internal Clinic. Various existing WWW presentations dealing with heart and breath sounds and other relevant investigations have been searched and listed.

**Discussion:**

Experience and feedback from other projects of this type confirm that a rather elaborate logical and technical construction of multimedia textbooks is rewarded by a good acceptance by both students and teachers. Good access to Internet, sufficient for multimedia transfers, however, is a necessary prerequisite. Internal propedeutics is a very suitable field for internet-based multimedia textbooks: instant access to audio and video recordings is much welcome in development of clinical skills. The project is supported by a grant of the Czech Universities Development Fund.

